# Wish list: which biomarker studies in HAM/TSP should be funded by the God of all trial funds?

**DOI:** 10.1186/1742-4690-11-S1-O28

**Published:** 2014-01-07

**Authors:** Yoshihisa Yamano

**Affiliations:** 1 Department of Rare Diseases Research, Institute of Medical Science, St. Marianna University School of Medicine, Kawasaki, Japan

## 

Since the discovery of HAM/TSP, little progress has been made on developing treatments. As HAM/TSP usually progress slowly, it can take a long time to evaluate disease activity and decide on a course of treatment. Therefore, there is a dire need for biomarkers capable of predicting the rate of disease progression and “surrogate end-points” that enable new treatments to be assessed more rapidly and with greater accuracy in clinical trials. However, in order to validate surrogate end-points, it is necessary to conduct large-scale, randomized clinical trials – a very difficult feat for such a rare disease. Here we present a recent study on candidate prognostic biomarkers for HAM/TSP and discuss future research directions.

